# One for All, All for One: A Close Look at In-Resin Fluorescence Protocols for CLEM

**DOI:** 10.3389/fcell.2022.866472

**Published:** 2022-06-30

**Authors:** Xavier Heiligenstein, Miriam S. Lucas

**Affiliations:** ^1^ CryoCapCell, Le Kremlin-Bicêtre, Paris, France; ^2^ Scientific Center for Light and Electron Microscopy (ScopeM), ETH Zurich, Zurich, Switzerland

**Keywords:** correlative light and electron microscopy (CLEM), in-resin fluorescence, contrasting for electron microscopy, preserving fluorescence, resin embedding, vitrification, freeze-substitution

## Abstract

Sample preparation is the novel bottleneck for high throughput correlative light and electron microscopy (CLEM). Protocols suitable for both imaging methods must therefore balance the requirements of each technique. For fluorescence light microscopy, a structure of interest can be targeted using: 1) staining, which is often structure or tissue specific rather than protein specific, 2) dye-coupled proteins or antibodies, or 3) genetically encoded fluorescent proteins. Each of these three methods has its own advantages. For ultrastructural investigation by electron microscopy (EM) resin embedding remains a significant sample preparation approach, as it stabilizes the sample such that it withstands the vacuum conditions of the EM, and enables long-term storage. Traditionally, samples are treated with heavy metal salts prior to resin embedding, in order to increase imaging contrast for EM. This is particularly important for volume EM (vEM) techniques. Yet, commonly used contrasting agents (e.g., osmium tetroxide, uranyl acetate) tend to impair fluorescence. The discovery that fluorescence can be preserved in resin-embedded specimens after mild heavy metal staining was a game changer for CLEM. These so-called in-resin fluorescence protocols present a significant leap forward for CLEM approaches towards high precision localization of a fluorescent signal in (volume) EM data. Integrated microscopy approaches, combining LM and EM detection into a single instrument certainly require such an “all in one” sample preparation. Preserving, or adding, dedicated fluorescence prior to resin embedding requires a compromise, which often comes at the expense of EM imaging contrast and membrane visibility. Especially vEM can be strongly hampered by a lack of heavy metal contrasting. This review critically reflects upon the fundamental aspects of resin embedding with regard to 1) specimen fixation and the physics and chemistry underlying the preservation of protein structure with respect to fluorescence and antigenicity, 2) optimization of EM contrast for transmission or scanning EM, and 3) the choice of embedding resin. On this basis, various existing workflows employing in-resin fluorescence are described, highlighting their common features, discussing advantages and disadvantages of the respective approach, and finally concluding with promising future developments for in-resin CLEM.

## 1 Introduction

The term “correlative microscopy” refers to the application of two or more imaging techniques to an identical sample, and at the exact same position, with the aim to combine the benefits of each respective technique. It has become state of the art technology in biomedical research (Su et al., 2010; Caplan et al., 2011; Jahn et al., 2012; Loussert Fonta and Humbel, 2015; Howes et al., 2018). Combining information from different methodologies to get a holistic understanding of the object of investigation is a fundamental concept in research, and was consequently adopted by microscopists soon after the advent of the transmission electron microscope (TEM). As early as 1944, Dubin and Sharp investigated the effect of sample preparation for TEM on the size of *B. megatherium* by means of correlating light microscopy (LM) images of air dried bacteria with TEM micrographs of the very same specimen (Dubin and Sharp, 1944). In doing so, they stumbled across the two major challenges of correlative microscopy: 1) how to prepare a sample such that it is suitable for all projected imaging techniques, and 2) how to relocate a region of interest (ROI) previously chosen with one microscopy method for correlation in the following, usually higher resolving microscope? And while science has since witnessed immense further developments of both, microscopes and sample preparation methods, these two challenges remain. Although the two topics are not fully unrelated, it would go beyond the scope of this review to discuss both, and we will focus on the former aspect of sample preparation.

Light and electron microscopy are routinely used in the field of life sciences. LM is an indispensable tool for biomedical research due to its time-resolving imaging capabilities and its ability to visualize individual proteins by means of immunofluorescence, or genetically encoded tagging (Giepmans et al., 2006). Electron microscopy (EM) perfectly complements LM, inasmuch as it visualizes a structure of interest at the nm-scale, and within its ultrastructural context. Consequently, the majority of correlative microscopy applications are variations of what has become known as “CLEM,” i.e., correlative light and electron microscopy. Countless variations of the concept are possible, allowing researchers to tailor a solution for basically every question to be addressed by means of microscopy, be it on the cellular level (Brown et al., 2009; Buser, 2010), for smaller organisms (Kolotuev et al., 2012; Goetz et al., 2015), tissue specimens (Wilke et al., 2008; Höhn et al., 2015), plants (Jahn et al., 2012; Bell et al., 2013) and animals (Karreman et al., 2014), at room temperature or cryogenic temperatures (Verkade, 2008; Yahav et al., 2011), for two-dimensional (Polishchuk et al., 2000; Giepmans et al., 2005; Keene et al., 2008) or three-dimensional (3D) imaging (Lucas et al., 2012; Wacker et al., 2015; Fermie et al., 2018). Indeed, there are so many possibilities, reflected by an ever increasing number of publications in the past decade, that recently an entire book chapter was dedicated to proposing guidelines to help researchers choose a suitable approach to CLEM (Scher and Avinoam, 2021).

**FIGURE 1:**
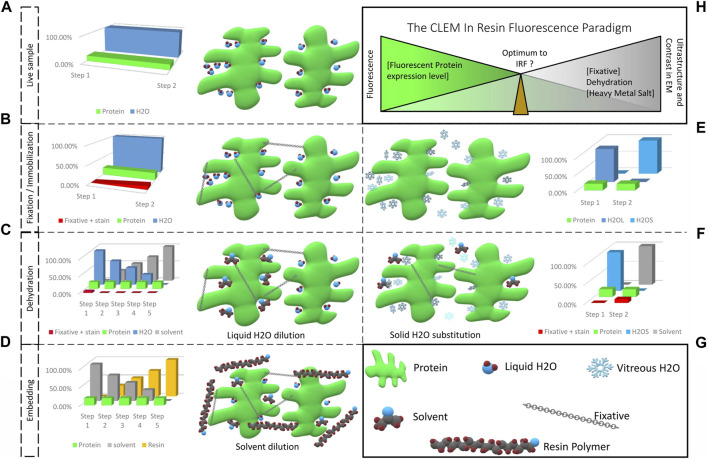
From a living fluorescent biological sample to an embedded specimen for light and electron microscopy, pitfalls and trade-offs. **(A)** Living matter is essentially composed of water and proteins, lipids and carbohydrates. Protein functions are related to their structural shape, supported by several scaffolding proteins. **(B)** During chemical fixation the sample is immobilized by cross-linking. The fixative, mostly aldehyde compounds, creates bonds between proteins, lipids and carbohydrates. Diffusion and fixation efficiency is affected by the sample’s density and the pH of local sub-compartments. **(C)** In the following dehydration in increasing concentrations of solvent, unbound fixative is progressively eluted together with the water bound in the sample. **(D)** The solvent is then progressively replaced by resin and polymerization is initiated (by heat or UV polymerization). Depending on the type of resin, chemical bonds can be formed between sample and resin, which provide complementary fixation. **(E)** Physical fixation, on the other hand, instantaneously and homogeneously immobilizes the entire sample, going from a hydrated living state (H_2_OL) to a cryo-immobilized state (H_2_OS) in a matter of milliseconds. Ice crystal growth resulting in damage of the ultrastructure is prevented and the water molecules remain in place. **(F)** The vitrified specimen is dehydrated in solvent at −90°C. The solvent substitutes the solid water, molecule by molecule. If chemical fixatives are added, these are simultaneously diffused into the specimen. These fixatives become active only above −60°C. **(G)** Graphical legend. **(H)** The CLEM in-resin fluorescence paradigm. Moving from the living, fluorescent sample to the ideal fixed electron microscopy material inevitably causes fluorescent protein quenching. The ideal IRF protocol is a compromise between acceptable fluorescence loss to identify the protein of interest after resin embedding, and adequate ultrastructure preservation and imaging contrast to achieve exploitable EM images.

EM requires high vacuum conditions to allow the electrons to travel from the source to a target detector, en route interacting with the sample. This imaging environment triggers a couple of pre-requisites for imaging hydrated biological material, in order to avoid sublimation of water and thus damage the specimen. To observe the material in close to physiological conditions, water (free, and partially also bound) must either be solidified, i.e., vitrified and imaged under cryogenic conditions, or removed. Specimen integrity has to be preserved under controlled conditions, e.g., by critical point drying (Bray, 2000), or ultimately by replacing vitrified water with a vacuum resistant substitute, i.e., embedding in resin. For the latter case several aspects are to be considered:

- Water represents a major constituent of biological material (Levitt and Gaudino, 1950; Reinoso et al., 1997; Ling, 2004). Its removal must be carried out carefully to avoid deformations or collapse of hydrated structures (Figure 1A). To turn the biological material into a vacuum resistant object, water is commonly substituted by a solvent, which in turn is substituted by a liquid resin, which is finally polymerized to form a solid sample.
- To maintain the ultrastructure during water substitution, the specimen needs to be stabilized, either by chemical fixation to create bonds between proteins, carbohydrates and lipids, or by maintaining the specimen in a frozen state after physical fixation, i.e., vitrification, such that the biological material is stabilized until its solidification by the resin.
- Finally, biological structures are mainly composed of molecules containing light elements, such as carbon (C, 12 g/mol), hydrogen (H, 1 g/mol), oxygen (O, 16 g/mol), and nitrogen (N, 14 g/mol), which renders them largely electron transparent. What is more, embedding resins consist of hydrocarbons, i.e., the identical light elements (C, H, O), and it is therefore hard to differentiate between biological material and surrounding embedding resin. To compensate for this lack of contrast, decoration with heavier atoms is commonly applied, e.g., using uranyl acetate or osmium tetroxide, to locally stop or deviate electrons from their trajectory and thus improve the resulting EM image.

This review summarizes the main strategies deployed over EM history for imaging biological material at room temperature. Cryogenic EM represents an independent research area and as such is not topic of this review. Consequently, we will focus here on the basic principles of sample fixation, contrast enhancement and resin embedding, particularly with regard to multimodal imaging strategies such as CLEM.

## 2 Sample Immobilization, Dehydration and Embedding: A Pre-Requisite for Electron Microscopy With Consequences

The first step toward rendering a specimen suitable for EM is immobilization, either chemically, or physically. This is followed by dehydration. After chemical fixation, dehydration can be performed either at room temperature, or while progressively lowering the temperature (Carlemalm et al., 1985; Robertson et al., 1992), depending on the embedding resin to be used. Physical fixation is followed by dehydration at temperatures below the melting point of water in order to prevent recrystallization of vitrified water, so-called freeze-substitution (FS). Contrast enhancement, as well as staining or labelling steps can be included at virtually every step of the process, as discussed later.

Infiltration with aldehyde compounds is the most commonly used standard for chemical fixation (Hayat, 1981). Aldehydes react with proteins, and to a certain extent, also with lipids, to create carbonyl bonds, which cross-link and thus preserve cellular ultrastructure (Figure 1B). Several factors influence the cross-linking reaction and with that the quality of the resulting fixation: temperature, pH, type and concentration of the applied aldehyde compound, optimal agitation and duration. The fixative locally affects the pH of the specimen (and is influenced by local pH, too), disturbing the ionic balance and affecting osmolality (Murk et al., 2003). Fixation at lower temperature generally reduces formation of artefacts, such as extraction of cellular components, granularity of the cytosol, or volume changes (Collins et al., 1977; Murk et al., 2003). However, infiltration speed and quality can be negatively affected at low temperature, e.g., for dense samples, while perfusion at room temperature is faster than in the cold (Hayat, 1981). Agitation additionally improves perfusion. Glutaraldehyde proved the most efficient and reliable, and most widely applicable aldehyde compound for fixation of biological specimens. It preserves ultrastructure best and causes the least conformational protein changes. Formaldehyde penetrates specimen faster than glutaraldehyde, but its reactions with proteins are reversible and it can be removed by washing with water (Hayat, 1981; Newman and Hobot, 1993). Therefore, combinations of both aldehyde types are widely used to combine the positive effects of speed and cross-linking quality (Karnovsky, 1965). Glutaraldehyde is commonly used at a concentration of 0.5%–2%, and formaldehyde at 0.5%–4%. Higher concentrations may cause severe shrinkage (Hayat, 1981). These aspects have to be carefully balanced and adapted to the respective specimen type.

With regard to preservation of antigenicity or fluorescence, formaldehyde is to be preferred over glutaraldehyde. Formaldehyde fixation preserves antigenicity and fluorescence when applied in concentrations up to 5% (Betzig et al., 2006; van Rijnsoever et al., 2008; Brown and Verkade, 2010; Hodgson et al., 2014). Glutaraldehyde on the other hand causes auto-fluorescence (excitation 540 nm, emission 560 nm). However, this can be quenched by sodium borohydride without quenching the target fluorescence of a fluorescent protein (Tagliaferro et al., 1997; Clancy and Cauller, 1998). The auto-fluorescence of aldehyde-based fixatives can even be favourably employed, as sole source of fluorescence in biopsies to navigate and choose a ROI at the LM level prior to targeted EM (Prior et al., 1999).

Following fixation, the sample is dehydrated by progressively diluting the contained water into a solvent (most commonly used: ethanol or acetone) of increasing concentration (from 25% to 100%; Figure 1C). Primarily, free water in the sample is diluted, and the water serving as a scaffolding for proteins, is progressively replaced by solvent molecules. As solvent and water molecules differ in size and properties, the proteins suffer some conformational changes that alter in part their properties (Buser and Walther, 2008). However, the initial fixation with aldehydes compensates partly for this deformation. Following the dehydration step, the solvent is then gradually replaced by resin, which is finally polymerized (Figure 1D). A similar suboptimal maintenance of the protein shape and properties as during dehydration can be assumed at this step. This can again be compensated for by the fixatives.

For physical fixation, the temperature of the sample is rapidly (>2 K/ms) lowered below the crystallization point of water (−135°C). This cryo-immobilizes the sample, and transfers the contained water into a glassy state, the so-called vitreous water (Figure 1E) (Moor, 1973; Riehle and Hoechli, 1973; Dubochet et al., 1987). In this state, water density and organization is most similar to its organization in liquid form, and therefore the sample is (to date) considered to be preserved closest to its native form. Maintaining the water in its solid form below the recrystallization point for water, prevents water sublimation and is the entry point to all cryo-EM workflows. For resin embedding, the vitrified sample is then subjected to FS. Comparable to room temperature dehydration, the sample is dehydrated in a solvent, but at temperatures below, or close to −90°C. At this temperature, the water molecules undergo conformational changes: from amorphous below −135°C, to crystalline cubic, until crystalline hexagonal form near −90°C. Frozen water remains solid until it is substituted, locally, by the solvent (Hippe-Sanwald, 1993). The dehydration process therefore does not cause extreme movements of water, which could impact the ultrastructure. As proteins and nucleic acids are insoluble in organic solvents, replacing water by solvents will result in aggregation of biological macromolecules. This aggregation cannot be avoided, but it can be limited in size, accounting for the superior ultrastructural preservation of FS compared to chemical fixation. This enables reliable volumetric analyses or measurements. Removing the hydration shell of proteins does not cause shrinkage, but potentially causes conformational changes. However, this effect can be limited at lower temperatures (Kellenberger, 1987). Replacement of the solvent and resin infiltration can finally be done at temperatures suitable for the resin of choice (Figure 1D).

Especially with regard to preserving fluorescence of genetically encoded labels, protein collapse or denaturation has to be strictly avoided (as discussed in detail in chapter 5). Prolonged exposure to acetone during dehydration was identified as potential reason for fluorescence loss, which was addressed successfully with significantly shortened FS-protocols (McDonald and Webb, 2011; Peddie et al., 2014). Acetone is often preferred over ethanol as dehydration medium during FS. It is described to be less extracting on the cytoplasm and a mild fixative (Weibull and Christiansson, 1986). However, it can impede polymerization of acrylic resins. For optimal results, a transition from acetone to ethanol prior to resin infiltration during FS is recommended (Monaghan et al., 1998; Watanabe et al., 2011). To further optimize ultrastructure preservation, fixatives can be added to the solvents in low concentrations. As aldehydes are estimated to become active only above −60°C (Wild et al., 2001; Buser and Walther, 2008), they can be considered inactive and insensitive to pH at the start of the substitution process (Figure 1F; Figure 1G). They are homogeneously distributed in the sample together with the solvent. Activation by slowly raising the temperature in the course of the FS enables the use of much lower concentrations of fixatives. Thus, artefacts associated with chemical fixatives, such as extensive aggregation of bio-macromolecules, or shrinkage can be minimized.

## 3 Choice of the Resin to Preserve Fluorescence

In addition to the aforementioned impact of fixation and dehydration on the specimen, the nature of the resin is also of importance for preserving fluorescence. Embedding in epoxy resins, such as Epon, Durcupan or Spurrs, offers optimal structural preservation (Luft, 1961; McDonald, 2007). But these resins are cured at high temperatures, i.e., above 50°C, which potentially causes protein denaturation (Lepock, 1997). Moreover, they form chemical bonds with nucleophiles present in proteins, which also results in denaturation of proteins. This reduces antigenicity or, in the case of fluorescent proteins, the ability to fluoresce (Causton, 1985; Matsko and Mueller, 2005). On the other hand, it improves the homogeneity of the sample by forming interpenetrating polymer networks, linking tissue and resin. This accounts for the excellent cutting property of these resins (Luft, 1961; Newman and Hobot, 1993). What is more, epoxy resins exhibit an auto-fluorescence, predominant in the “green” range of light (approx. 500–550 nm), which coincides with the maximum emission wavelength of commonly used fluorophores, such as green fluorescent protein (GFP) (Zhou et al., 2017; Fu et al., 2020).

Acrylic resins, e.g., HM20, K4M, GMA, LR White, or R221, on the other hand, mostly react with themselves and form bonds with proteins at sulphydryl and thioether sites only (Carlemalm et al., 1985; Causton, 1985; Kellenberger et al., 1987). Biomolecules are not incorporated into the polymer, and therefore, acrylic resins form weaker links to the sample material (Keene et al., 2008). The strength of these links, as well as hardness and brittleness of the resin depend on the length of the composing monomer chains (short with HM20 and K4M, longer with R221), and the amount of applied crosslinker. As bonds are formed only at the ends of each monomer chain, longer monomer chains will result in a lower crosslink density (Carlemalm et al., 1982). What is more, these resins have a better tolerance of residual water in the embedded material. If a specimen is not fully dehydrated, the remaining water content maintains the scaffolding for proteins, and thus preserves their conformation (Walther and Ziegler, 2002; Buser and Walther, 2008). In order to further enhance the stability of the biological material during the embedding procedure, most acrylic resins are designed for application at low temperature, i.e., below approximately −20°C (Carlemalm et al., 1982). An important aspect to be considered with respect to the preservation of fluorescence or antigenicity is the pH of the resin. Several acrylic resins are too acidic when used in the standard formulation, and hence can cause substantial loss of fluorescence (Watanabe et al., 2011; Zhou et al., 2017). Nevertheless, proteins can be sustained in, or close to the natural, hydrated state, which prevents conformational changes. They are therefore more accessible for antigen detection (Berryman and Rodewald, 1990; Hayat, 2002). Additionally, the electron mobility in (fluorescent) proteins is maintained, so that excitation and fluorescence emission remain possible (Nixon et al., 2009). This makes acrylic resins the better choice for fluorescence preservation.

## 4 Contrast Enhancement: A Necessary Evil?

The last important step for EM on resin-embedded materials is to enhance the contrast of the structures of interest. Organic materials consist mainly of low atomic number elements (such as carbon, hydrogen, oxygen), and consequently such specimens do not generate much electron scattering contrast in the EM. What is more, embedding resins are composed of similar elements with low electron density. And although it is desirable for the embedding resins to produce little or no background contrast, this underlines the necessity of contrast enhancement, to visualize the biological specimen and distinguish it from the resin. This is generally achieved by staining with heavy metal compounds, with more or less specific affinity for proteins or lipids. Contrast enhancement steps can be carried out during, or after, fixation and (or) during dehydration. It is interesting to observe that the literature was very rich between the 1950s and 1980s with efforts to highlight specific cellular compartments according to their chemistry. Various heavy metal compounds were investigated, with varying success. Hayat (1993) provides an excellent collection of these findings. Of these tested staining agents, only a few prevail and are used to date, the most common being uranyl acetate and osmium tetroxide. At that time, EM images were recorded on negative film and the target structures were identified on the fluorescent screen of the EM. Since the advent of more sensitive digital cameras, image recording has become fast and straightforward in operation. Corrections can be made live on the computer screen in order to optimize focus and contrast. This, among other things, allowed researchers to reduce the efforts put into optimizing staining procedures and consequently, the vast majority have returned to standard protocols based on uranyl acetate and osmium tetroxide. However, osmium tetroxide is well-known to impair fluorescence (Watanabe et al., 2011), this effect being predominant with fluorophores emitting in the “green” range of light (approx. 500–550 nm). Fluorophores emitting in the “red-to far-red” range (approx. 630–750 nm) are less susceptible, or even resistant to this impairment (Lucas et al., 2012; Lucas M. S. et al., 2017; Fu et al., 2020). Uranyl acetate was demonstrated to have a weak fluorescence, which can still be detected in resin-embedded samples (Biel et al., 2003; Tuijtel et al., 2017). And, although this effect can be neglected in the presence of strong fluorescent labels, it still impairs the signal-to-noise ratio and limits the detection of weak fluorescent signals.

In recent years, use of uranyl acetate is subject to tighter legislation, which triggered a new search for alternatives (Inaga et al., 2007; Yamaguchi et al., 2010; Nakakoshi et al., 2011; Hosogi et al., 2015; Kuipers and Giepmans, 2020). However, as most of these staining compounds are insoluble in organic solvents, their application is reduced to room temperature embedding procedures and poststaining of resin sections. An alternative for FS applications remains to be discovered, and uranyl acetate remains the gold standard.

A noteworthy aspect of any contrasting procedure is the fact that the employed heavy metal compounds simultaneously act as fixatives (Sabatini et al., 1963; Ginsburg and Wolosin, 1979; Erickson et al., 1987; Hayat, 1993). This reinforces the primary fixation, but concomitantly reduces a proteins’ ability to fluoresce (Berryman and Rodewald, 1990; Nixon et al., 2009). Hence, consensus has grown around the need to minimize fixatives as well as contrasting agents in order to preserve fluorescence after resin embedding (Biel et al., 2003; Nixon et al., 2009; Kukulski et al., 2011; Lucas et al., 2012; Peddie et al., 2014; Delevoye et al., 2016; Baatsen et al., 2021). For TEM applications, sensitive detection systems (EMCCD, CMOS and direct electron detectors) enable minimal use of uranyl acetate, at concentrations below 1% in FS protocols, yielding impressive image quality (Hawes et al., 2007). Unfortunately, improved detection sensitivity is not the most critical parameter for volume EM (vEM) approaches, such as serial blockface SEM, focused ion beam-SEM (FIB-SEM), or array tomography. Upon scanning the surface of a sample, electron charging builds up rapidly, compromising iterative imaging approaches. To compensate for the charging, the conductivity of *en bloc* samples can be increased by enhanced heavy metal staining (Deerinck et al., 2010). While this has the additional advantage of increasing the contrast in the EM, it entails the complete loss of fluorescence. The recent development of a focal charge compensation (Deerinck et al., 2018), significantly reduces the need for such highly (over- ?) stained samples. Reducing the heavy metal salt loading again offers the possibility to preserve the fluorescence.

## 5 One sample for all Imaging Modes or the Ideal CLEM Sample: Myth or Reality?

The basic concept of CLEM to investigate the exact same structure with LM and EM was addressed by countless approaches (Table 1). Many of them rely on staggered sample preparation protocols, i.e., performing LM and EM separately, one after the other, in order to maintain optimum conditions for both LM and EM (Mironov et al., 2000; Sims et al., 2006; van Rijnsoever et al., 2008; Knott et al., 2009; Lucas M. S. et al., 2017). However, the ideal sample for CLEM would be suitable for all imaging modes at once, i.e., the preparation should balance preservation of fluorescence labels and heavy metal staining, while avoiding structural changes to the specimen Figure 1H. The emergence of integrated light and electron microscopes (Timmermans and Otto, 2015) even made this a requirement.

**TABLE 1:** Fluorescent labels for IRF.

Label name	Demonstrated for	Highlighting	Application		References
			In the freeze-substitution	On-section labeling	
Non-specific labels					
1.8 ANS	Mammalian tissue	Collagen and/or elastic fibers	✓		Biel et al. (2003)
Acridine orange	Mammalian tissue	Nuclei, cytoplasm	✓		Biel et al. (2003)
	Cultured cells, bacteria	Cytoplasm, nuclei	✓		Lucas et al. (2012)
Bodipy 560	Mammalian tissue	Nuclei, cell membranes	✓		Biel et al. (2003)
DCVJ	Mammalian tissue	Nuclei, collagen and/or elastic fibers	✓		Biel et al. (2003)
DiD	Mammalian tissue	Lipophilic domains, cell membranes	✓		Biel et al. (2003)
	Cultured cells	Lipophilic domains, cell membranes	✓		Lucas et al. (2012)
DiIC_18_	Mammalian tissue	Lipophilic domains, cell membranes	✓		Biel et al. (2003)
	Cultured cells	Cell membranes	✓		Lucas et al. (2012)
DiOC_6_	Mammalian tissue	Cytoplasm (lipophilic domains)	✓		Biel et al. (2003)
Nile blue sulfate	Mammalian tissue	Nuclei, cytoplasm, collagen, elastic fibers	✓		Biel et al. (2003)
	Model organisms, e.g., *C. elegans*	Cytoplasm, cell membranes	✓		Lucas et al. (2012)
	Cultured cells	Nuclei, cytoplasm, cytoskeleton*	✓		Lucas et al. (2012)
Nile red	Cultured cells	Nuclei, cytoplasm	✓		Lucas et al. (2012)
Oregon Green	Mammalian tissue	Nuclei, collagen, elastic fibers	✓		Biel et al. (2003)
Safranin O	Mammalian tissue	Nuclei, cytoplasm, collagen, elastic fibres	✓		Biel et al. (2003)
	Cultured cells	Nuclei, cytoplasm	✓		Lucas et al. (2012)
	Model organisms, e.g., *C. elegans*	Nuclei, cytoplasm, cell membranes	✓		Lucas et al. (2012)
	Plant tissue	Entire tissue, unspecific	✓		Lucas et al. (2012)
Syto 24	Cultured cells	Entire cell, unspecific	✓		Lucas et al. (2012)
Syto 83	Cultured cells	Entire cell, unspecific	✓		Lucas et al. (2012)
Sytox Green	Cultured cells	Entire cell incl. membranes, unspecific	✓		Lucas et al. (2012)
Tannin	Mammalian tissue	Nuclei	✓		Biel et al. (2003)
Uranyl acetate	Any type of biological specimen	Entire tissue or cell	✓	✓ Note the temperature dependency!	(Biel et al., 2003; Tuijtel et al., 2017)
Specific labels					
Dapi	Any type of biological specimen	Nuclei		✓	
Hoechst	Any type of biological specimen	Nuclei		✓	Delevoye et al. (2016)
Phalloidin Alexa 488	Cultured cells and mammalian tissue	Cytoskeleton	✓		Lucas et al. (2012)
Genetically encoded tags					
	**Standard IRF**	**Super-resolution LM**	**References**		
Citrine	✓		Watanabe et al. (2011)		
GFP/mGFP	✓	✓	(van Rijnsoever et al., 2008; Nixon et al., 2009; Kukulski et al., 2011; Johnson et al., 2015; Delevoye et al., 2016; Bissig et al., 2017; Peddie et al., 2017; Burel et al., 2018; Ripoll et al., 2018)		
mCherry	✓	✓	(Höhn et al., 2015; Ripoll et al., 2018)		
mEos-derivatives		✓	(Watanabe et al., 2011; Paez-Segala et al., 2015; Kopek et al., 2017; Fu et al., 2020)		
mRuby2		✓	Johnson et al. (2015)		
mVenus		✓	Johnson et al. (2015)		
YFP	✓		(Heiligenstein et al., 2014; Delevoye et al., 2016)		

Table adapted from Lucas et al. (2012) with permission of Elsevier Ltd.

So-called “in-resin fluorescence” (IRF) protocols, were developed to meet this requirement. However, as pointed out above, preserving fluorescence even after resin embedding is a complex endeavour. Virtually every single one of the four challenges, namely fixation, dehydration, contrasting and resin embedding may impair the capacity of fluorescent proteins or other labels to be excited and emit fluorescence. What the majority of IRF protocols have in common: they base 1) on cryo-fixation, e.g., HPF, 2) low-temperature, and in particular incomplete dehydration, in order to preserve the hydration shell of the proteins, and 3) a minimal use of contrasting agents for EM, with osmium tetroxide even completely omitted in most cases. This is followed by 4) embedding in acrylic resins which have a low interaction with the sample.

### 5.1 Non-Specific Labelling for a Morphological Ultrastructure Identification

**FIGURE 2:**
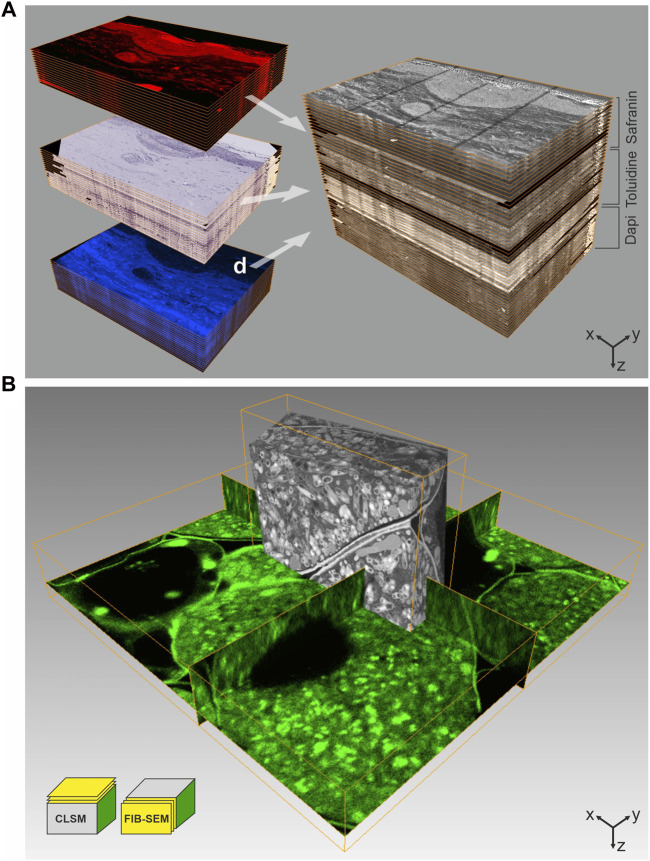
CLEM using IRF samples. **(A)** Array tomography. 100 nm thick serial sections of a human skin biopsy, high-pressure frozen, freeze-substituted with uranyl acetate, osmium tetroxide and Safranin O, and embedded in HM20 were mounted on ITO-coated glass coverslips. This approach allows multiple on-section labeling to complement IRF. The top subset was imaged by fluorescence LM without further staining, using the Safranin O signal introduced during FS, the middle subset shows on-section staining with Toluidine, and the subset of sections was stained with Dapi. Following LM, all sections were imaged by SEM at 2 kV using in-lens SE detection. Combination of all images yields a volume that comprises multiple levels of information, ideal for in-depth characterization of complex biological structures. Volume size (x-y-z): LM stack: 321.0 × 221.5 × 5.1 µm³, SEM stack: 226.4 × 170.0 × 5.1 µm³. **(B)** 3D CLEM of root nodule from mung bean (*Vigna radiata*), colonized with nitrogen-fixing bacteria (*B. japonicum*). 200 µm thick sections were high-pressure frozen after degassing in 1-hexadecene, followed by FS and embedding in HM20. The FS-medium contained uranyl acetate, osmium tetroxide and Acridine orange for fluorescent labeling of the bacteria. En bloc CLSM was performed to identify a ROI, which was subsequently targeted by FIB-SEM. The CLSM and FIB-SEM volumes were merged using the Amira software package. The imaging plane of FIB-SEM is perpendicular to that of CLSM. Volume size (x-y-z): CLSM 57.2 × 57.2 × 9.0 µm³, FIB-SEM 21.8 × 6.2 × 15.9 µm³. Figure adapted from Lucas et al. (2012) with permission of Elsevier Ltd.

One of the first approaches to allow LM and EM on an identical sample was designed by Biel et al. (2003). They employed the auto-fluorescence of uranyl acetate in resin-embedded tissue for *en bloc* imaging using confocal laser scanning microscopy (CLSM). A recent work further demonstrated the benefit of uranyl acetate auto-fluorescence, which was observed to increase significantly at low temperatures, i.e., below −100°C. This auto-fluorescence detected in resin sections could be matched precisely with the contrast obtained by TEM, facilitating a “on-section” image registration (Tuijtel et al., 2017). Biel at al. (2003) also included fluorescent dyes in the staining and dehydration cocktail during FS. In this way, high-pressure frozen samples could be fluorescently stained and contrasted with uranyl acetate prior to resin embedding. The resulting specimens were equally suitable for LM and EM, and combined the excellent structural preservation provided by high-pressure freezing (HPF) with staining for *en bloc* CLSM. Despite the labelling not being specific, this technique allowed a detailed overview of complex tissue samples using LM. The LM map was then used to select a ROI within the resin-embedded specimen to facilitate targeted ultramicrotomy for TEM. This approach paved the way for what is today referred to as “in-resin fluorescence protocols.” Later, we advanced this protocol for FIB-SEM imaging (Figure 2B), using the fluorescent labelling as guidance to identify the ROI for targeted FIB-milling (Lucas et al., 2012). While Biel et al. (2003) had omitted any heavy metal salts except for uranyl acetate, we again included osmium tetroxide during FS, in order to enhance imaging quality and contrast in FIB-SEM. Despite its well-known interference with fluorescence, we found only minimal changes in fluorescence signal distribution and intensity of the tested dyes, when removing the osmium from the FS-cocktail at −50°C. Still, the osmium staining did improve the visibility of cellular membranes at the EM level as compared to using only uranyl acetate. Even though this approach identifies a ROI based on the morphology of fluorescent features rather than by specific labelling, it is easy to implement and can be applied to nearly any specimen.

Besides their simplicity, a main benefit of these approaches is the maintenance of specimen integrity. Potential damage to the ultrastructure, e.g., due to permeabilisation to introduce stains, are completely avoided, because all labels or stains are applied only after HPF. The observation of the entire sample allows the identification of a ROI prior to targeted ultramicrotomy, or FIB-SEM milling and imaging. Karreman et al. developed a similar strategy of global 3D mapping of the readily embedded sample, by using soft-X-ray imaging to relocate a ROI previously identified in a living animal. However, this approach did not allow the preservation of in-resin fluorescence (Karreman et al., 2016; Karreman et al., 2017).

### 5.2 Genetically Encoded Labels

Efficient identification of a structure of interest is essential for CLEM. Specific labelling and precise relocation of the labelled structure across different imaging modalities therefore becomes the pivot point. As discussed above, labelling may be altered during sample preparation for EM. In its early stages of discovery, GFP demonstrated a good tolerance to fixatives such as formaldehyde and glutaraldehyde (Chalfie et al., 1994; Ward, 2006), although the latter fixative can cause imaging difficulties due to its high auto-fluorescence. GFP was reported to denature when dehydrated in pure, dried organic solvents (Ward, 2006). As a result, its fluorescence is quenched, with the extent of the quenching depending on the grade of dehydration. Luby-Phelps et al. (2003) showed on aldehyde-fixed GFP-tagged photoreceptors in the retina of zebrafish embryo that retaining a specific fluorescence label is possible after incomplete dehydration in organic solvents, and resin embedding. They completely omitted heavy metal stains commonly used for EM, and embedded the samples in LR White. Using one-µm thick sections, they could for the first time demonstrate fluorescence detection from within the resin-embedded material. This first proof that GFP fluorescence can indeed survive EM sample preparation at room temperature was a major breakthrough and the protocol was later successfully adapted to maize tissue (Rizzo et al., 2016).

Independently, Walther and Ziegler demonstrated that FS of vitrified specimens in solvents containing up to 20% water resulted in improved ultrastructural preservation, and especially enhanced the visibility of biological membranes (Walther and Ziegler, 2002). FS in strictly dried solvents, on the other hand, resulted in significantly reduced contrast and definition of membranes. Putting two and two together, Nixon et al. (2009) made use of this incomplete dehydration to combine the excellent ultrastructural preservation of HPF/FS with preservation of GFP fluorescence in HM20 embedded zebrafish embryo samples. This major discovery of the ability to preserve genetically encoded fluorescence after HPF and FS, including EM staining, opened a new era in correlative microscopy. IRF was used with high accuracy and relatively high throughput to localize endocytosis in yeast (Kukulski et al., 2011). Since then, the technique has been applied to a wide range of biological questions. IRF is a highly versatile tool, and can be combined with the follow-up EM method of choice. A staggered approach employed consecutive sections, starting with relatively thick sections (i.e., 300 nm) for LM in order to improve detection of IRF and target the structure of interest for TEM, which was then performed on 50–70 nm thin sections (Delevoye et al., 2016). Bypassing the consecutive sectioning approach and directly analysing the thicker sections by electron tomography, moved IRF applications into the third dimension (Kukulski et al., 2012; Bissig et al., 2017). The combination with live cell imaging prior to HPF makes IRF an even more versatile and powerful tool, enabling even the study of dynamic processes (Heiligenstein et al., 2014; Ripoll et al., 2018).

IRF was also employed in CLEM approaches based on vEM, to make use of the larger field of view and stable, automated acquisition methods for volume data (Peddie and Collinson, 2014; Titze and Genoud, 2016). The fluorescence signal can e.g., be used to screen serial sections, so-called arrays, and identify a ROI for high-resolution SEM, a technique dubbed “array tomography” (Micheva and Smith, 2007; Wacker and Schroeder, 2013). To reduce charging artefacts during SEM imaging, the arrays are mounted onto conductive supports such as silicone wafer, or indium tin oxide (ITO)-coated glass. The latter being transparent, makes it advantageous for LM (Lucas et al., 2012). These arrays can be stored and re-investigated, as well as further stained or contrasted, making array tomography highly suited to localize rare cellular structures or events in complex tissue samples (Micheva et al., 2010; Oberti et al., 2010; Burel et al., 2018; Lane et al., 2022). Analogous to approaches described in the previous subchapter, Höhn et al. (2015) detected mCherry-labeled virus particles by *en bloc* CLSM, prior to acquisition of volume data by FIB-SEM. The development of the “ultraLM” and “miniLM” allows fluorescence detection from within a resin block to be directly combined with ultramicrotomy applications (Brama et al., 2016). The “ultraLM” is directly mounted onto a standard ultramicrotome to locate and follow fluorescent target structures while trimming. The “miniLM,” an add-on to a serial-blockface SEM, presents an integrated solution for consecutive detection of fluorescence signal and recording of blockface scans for vEM data in a single instrument. Another highly sensitive microtome-integrated microscope solution monitors the fluorescence in sections floating in the diamond knife boat directly after cutting. Here, hydration of the sections additionally promotes the fluorescence, which is used to identify sections of interest for further analysis (Lemercier et al., 2017).

### 5.3 Non-Genetically Encoded Specific Labels

For historical reasons, GFP, RFP and mCherry are the most commonly used fluorescent proteins, although they often show a low quantum yield and a low brightness (Wall et al., 2015; Prangsma et al., 2020). Fluorescent dyes, such as i.e., Alexa dyes on the other hand are very bright, and tolerate fixation, dehydration and resin embedding very well. Associating them to a specific and physiological label yields a reliable tool for CLEM. Delevoye et al. (2016) for instance, added a transferrin protein coupled to an Alexa-dye to the culture medium of HeLa cells prior to HPF and subsequent processing. The internalized transferrin labelled the endocytic pathway homogeneously and produced a very bright fluorescence signal in resin sections. This served as a guide to identify the most promising phenotype, and locate the fainter fluorescent signal obtained from a genetically encoded mutant. As another example, phalloidin coupled to Alexa dyes can be included in the FS cocktail for pre-embedding specific labelling of actin (Lucas et al., 2012).

It is noteworthy, that besides using genetically encoded tags, additional on-section staining with other specific labels can be performed to complement IRF and aid navigation between LM and EM (Figure 2A). Nucleic stains such as DAPI or Hoechst can be easily applied on sections, both on SEM and TEM sample supports. These specific, intercalating dyes can still interact with chromatin in resin-embedded samples, and yield a bright and stable fluorescent signal (Lucas et al., 2012; Delevoye et al., 2016; Lane et al., 2022).

### 5.4 Optical Super-Resolution Comes Into Play

With super-resolution LM becoming widely available, efforts were also made towards super-resolution CLEM to close the resolution gap between fluorescence LM and EM. Super-resolution LM can be well implemented on resin sections mounted on ITO coated glass slides as used for array tomography. However, this requires specialized labels, which remain photoactivatable even after resin embedding, and if possible also tolerate EM contrasting agents (Kopek et al., 2017). First approaches were made based on chemical fixation. Betzig et al. (2006) correlated photoactivated localization microscopy (PALM) and TEM using chemically fixed cryo-sections. Stimulated emission depletion (STED) microscopy and PALM on resin-embedded samples was then demonstrated by Watanabe et al. (2011), using the fluorescent proteins citrine and tdEos. Paez-Segala et al. (2015) reported the photoconvertible Eos fluorescent protein to fluoresce and photoconvert normally in samples treated with osmium tetroxide, and embedded in acrylic resin. Another variant of the “Eos protein family,” mEosEM, even tolerates embedding in epoxy resin (Fu et al., 2020). In-resin super-resolution microscopy for standard fluorescent proteins such as mGFP, mVenus and mRuby2 after cryo-fixation and FS, was established by Johnson at al. (2015). They showed that single molecule photo-switching can be detected from HM20-embedded specimens containing fluorescent proteins when mounting TEM sections in a glycerol-based mounting medium. With this single molecule localization microscopy approach they achieved a structural resolution of approx. 50 nm in fluorescence microscopy. That some fluorescent proteins show the blinking behaviour employed for single molecule localization microscopy also in dry mounted resin sections, and even under vacuum conditions was discovered by Peddie et al. (2017). *In vacuo* single molecule localization microscopy allowed them to take advantage of an integrated light and SEM setup to directly acquire both, super-resolution LM and SEM images on the same resin section. As the sample is not moved between the two imaging modalities, it allows a direct and precise overlay of the LM and EM images, and thus overcomes the problem of relocating a ROI after imaging in separate microscopes.

## 6 Future Perspectives

The major disadvantage of minimizing the heavy metal staining in order to preserve fluorescence in resin-embedded specimen is the compromise on EM imaging contrast, i.e., visibility of key features such as membranes. To overcome this, routine poststaining procedures, e.g., involving Reynold’s lead citrate and aqueous uranyl acetate, can be applied on sections to improve imaging contrast for TEM, or (serial) section SEM. It seems reasonable to add this step after fluorescence imaging, to avoid artefacts, caused by the auto-fluorescence of uranyl acetate. However, the penetration depth of poststaining agents into resin is limited (Hayat, 1993). Especially the commonly used uranyl acetate produces a detectable concentration gradient in depth already in sections of 300 nm thickness, while lead citrate showed a homogenous distribution throughout the section (Tranfield et al., 2014). This can have adverse effects especially for electron tomography applications. The recent improvements in TEM detector sensitivity may even make poststaining obsolete (Faruqi and McMullan, 2011; Clough et al., 2014; Fermie et al., 2020; Levin, 2021). For integrated microscopy solutions (Agronskaia et al., 2008; Liv et al., 2013; Brama et al., 2016), as well as for *en bloc* vEM applications, however, poststaining is impossible. Especially for vEM, imaging contrast remains a critical factor, as it mostly relies on the detection of backscattered electrons, and therefore requires contrasting by heavy metal compounds. Efforts are ongoing to improve those detection systems, too. Retarding (or deceleration) fields applied between electron objective lens and sample have been shown to improve imaging contrast in SEM and FIB-SEM, and therefore allow the reduction of heavy metal staining (Lane et al., 2021). Lately, Vos et al. (2020) have successfully applied a retarding (or deceleration) field in an integrated fluorescence and SEM setup.

Besides technological improvements, new labelling approaches are being explored. Moving away from fluorescent proteins, or other classical fluorescent labels for LM, several attempts were made to utilize nano-diamonds and nano-crystals as probes for CLEM (Hemelaar et al., 2017; Keevend et al., 2020). These labels retain their fluorescence even after treatment with osmium tetroxide and resin embedding. Besides being fluorescent, and well detectable in the SEM *via* their back-scattered electron signal, they also exhibit cathodoluminescence, which enables a new form of integrated microscopy for simultaneous imaging. To date, these probes are quite large in size (40–70 nm), which limits their application due to artefacts associated with incorporation into cells. The strong fluorescent or cathodoluminescence signals of these probes, as well as those of quantum-dots (Giepmans et al., 2006), have the large advantage that they can be well detected also against the background-fluorescence of epoxy resins. This again opens up possibilities for room temperature embedding approaches.

Alternative EM stains, such as lanthanides, platinum blue or Oolong tea extract have been investigated as potential replacement for uranyl acetate (Sato et al., 2003; Inaga et al., 2007; Hosogi et al., 2015). And although so far none of these substances can fully replace uranyl acetate, some are routinely used for EM sample preparation (Kremer et al., 2015). Lately, a more promising candidate has been proposed: neodymium acetate (Kuipers and Giepmans, 2020). Even though neodymium is still a heavy metal compound and is insoluble in solvents, its full potential remains to be explored, also with respect to CLEM. In general, contrasting agents with low chemical activity are of interest, and uranyl acetate remains to date the best compromise, providing a gentle stabilization of proteins, and good imaging contrast, while exhibiting a low auto-fluorescence at room temperature.

With respect to the dehydration medium, acetone is commonly preferred over ethanol (Humbel and Schwarz, 1989; Monaghan et al., 1998; Wild et al., 2001; Walther and Ziegler, 2002; Biel et al., 2003; Giddings, 2003; Hawes et al., 2007). However, the lower melting point of ethanol (−117°C vs. −95°C for acetone), as well as the higher polarity of the solvent could be worth exploring with regard to the preservation of fluorescence (Walther and Ziegler, 2002; Buser and Walther, 2008).

Another addition to the IRF toolbox is the newly developed embedding resin R221 (Heiligenstein et al., 2021; Jansen et al., 2021). This acrylic based resin was developed to reduce electron charge accumulation at the surface of the resin block. Initial results demonstrate high EM imaging contrast, even with very low concentrations of uranyl acetate staining (0.05% UA, with the addition of 5% H_2_O), allowing preservation of bio-fluorescence, while displaying fine contrast at high resolution in EM.

Altogether, the last decade witnessed the emergence of several innovative tools to reduce the gap between LM and EM using resin-embedded samples. Imaging the exact same sample with multiple microscopy techniques bears such great scientific potential that multiple solutions were developed independently. In the coming years, it can be expected that the increasing demand for CLEM will see a combination of all these tools into one single pipeline to achieve high throughput, at high speed, and high accuracy analysis of multiplexed samples.

## References

[B1] Agronskaia A. V. Valentijn J. A. van Driel L. F. Schneijdenberg C. T. Humbel B. M. van Bergen En Henegouwen P. M. (2008). Integrated Fluorescence and Transmission Electron Microscopy. J. Struct. Biol. 164, 183–189. 10.1016/j.jsb.2008.07.003 18664385

[B2] Baatsen P. Gabarre S. Vints K. Wouters R. Vandael D. Goodchild R. (2021). Preservation of Fluorescence Signal and Imaging Optimization for Integrated Light and Electron Microscopy. Front. Cell Dev. Biol. 9, 737621. 10.3389/fcell.2021.737621 34977003 PMC8715528

[B3] Bell K. Mitchell S. Paultre D. Posch M. Oparka K. (2013). Correlative Imaging of Fluorescent Proteins in Resin-Embedded Plant Material. Plant Physiol. 161 (4), 1595–1603. 10.1104/pp.112.212365 23457228 PMC3613441

[B4] Berryman M. A. Rodewald R. D. (1990). An Enhanced Method for Post-Embedding Immunocytochemical Staining Which Preserves Cell Membranes. J. Histochem Cytochem. 38 (2), 159–170. 10.1177/38.2.1688894 1688894

[B5] Betzig E. Patterson G. H. Sougrat R. Lindwasser O. W. Olenych S. Bonifacino J. S. (2006). Imaging Intracellular Fluorescent Proteins at Nanometer Resolution. Science 313 (5793), 1642–1645. 10.1126/science.1127344 16902090

[B6] Biel S. S. Kawaschinski K. Wittern K.-P. Hintze U. Wepf R. (2003). From Tissue to Cellular Ultrastructure: Closing the Gap between Micro- and Nanostructural Imaging. J. Microsc. 212 (1), 91–99. 10.1046/j.1365-2818.2003.01227.x 14516366

[B7] Bissig C. Hurbain I. Raposo G. van Niel G. (2017). PIKfyve Activity Regulates Reformation of Terminal Storage Lysosomes from Endolysosomes. Traffic 18 (11), 747–757. 10.1111/tra.12525 28857423

[B8] Brama E. Peddie C. J. Wilkes G. Gu Y. Collinson L. M. Jones M. L. (2016). ultraLM and miniLM: Locator Tools for Smart Tracking of Fluorescent Cells in Correlative Light and Electron Microscopy. Wellcome Open Res. 1, 26. 10.12688/wellcomeopenres.10299.1 28090593 PMC5234702

[B9] Bray D. (2000). Critical Point Drying of Biological Specimens for Scanning Electron Microscopy. Methods Biotechnol. Supercrit. Fluid Methods Protoc. 13, 1716. 10.1385/1-59259-030-6:235

[B10] Brown E. Mantell J. Carter D. Tilly G. Verkade P. (2009). Studying Intracellular Transport Using High-Pressure Freezing and Correlative Light Electron Microscopy. Seminars Cell and Dev. Biol. 20 **,** 910–919. 10.1016/j.semcdb.2009.07.006 19660566

[B11] Brown E. Verkade P. (2010). The Use of Markers for Correlative Light Electron Microscopy. Protoplasma 244 (1-4), 91–97. 10.1007/s00709-010-0165-1 20524017

[B12] Burel A. Lavault M.-T. Chevalier C. Gnaegi H. Prigent S. Mucciolo A. (2018). A Targeted 3D EM and Correlative Microscopy Method Using SEM Array Tomography. Development 145 (12), dev160879. 10.1242/dev.160879 29802150

[B13] Buser C. (2010). Toward Sub-second Correlative Light and Electron Microscopy of *Saccharomyces cerevisiae* . Methods Cell Biol. 96, 217–234. 10.1016/s0091-679x(10)96010-x 20869525

[B14] Buser C. Walther P. (2008). Freeze-Substitution: The Addition of Water to Polar Solvents Enhances the Retention of Structure and Acts at Temperatures Around -60Â°C. J. Microsc. 230 (2), 268–277. 10.1111/j.1365-2818.2008.01984.x 18445157

[B15] Caplan J. Niethammer M. Taylor R. M. Czymmek K. J. Czymmek K. J. (2011). The Power of Correlative Microscopy: Multi-Modal, Multi-Scale, Multi-Dimensional. Curr. Opin. Struct. Biol. 21 (5), 686–693. 10.1016/j.sbi.2011.06.010 21782417 PMC3189301

[B16] Carlemalm E. Garavito R. M. Villiger W. (1982). Resin Development for Electron Microscopy and an Analysis of Embedding at Low Temperature*. J. Microsc. 126 (2), 123–143. 10.1111/j.1365-2818.1982.tb00362.x 7040669

[B17] Carlemalm E. Villiger W. Hobot J. A. Acetarin J.-D. Kellenberger E. (1985). Low Temperature Embedding with Lowicryl Resins: Two New Formulations and Some Applications. J. Microsc. 140 (Pt 1), 55–63. 10.1111/j.1365-2818.1985.tb02660.x 3912508

[B18] Causton B. E. (1985). Does the Embedding Chemistry Interact with Tissue. Scanning Electron Microsc. 4, 209–214. The science of biological specimen preparation for microscopy and microanalysis.

[B19] Chalfie M. Tu Y. Euskirchen G. Ward W. W. Prasher D. C. (1994). Green Fluorescent Protein as a Marker for Gene Expression. Science 263 (5148), 802–805. 10.1126/science.8303295 8303295

[B20] Clancy B. Cauller L. J. (1998). Reduction of Background Autofluorescence in Brain Sections Following Immersion in Sodium Borohydride. J. Neurosci. Methods 83 (2), 97–102. 10.1016/s0165-0270(98)00066-1 9765122

[B21] Clough R. N. Moldovan G. Kirkland A. I. (2014). Direct Detectors for Electron Microscopy. J. Phys. Conf. Ser. 522, 012046. 10.1088/1742-6596/522/1/012046

[B22] Collins V. P. Arborgh B. Brunk U. (1977). A Comparison of the Effects of Three Widely Used Glutaraldehyde Fixatives on Cellular Volume and Structure. A TEM, SEM, Volumetric and Cytochemical Study. Acta Pathol. Microbiol. Scand. A 85A (2), 157–168. 10.1111/j.1699-0463.1977.tb00413.x 403740

[B23] Deerinck T. Bushong E. Lev-Ram V. Shu X. Tsien R. Ellisman M. (2010). Enhancing Serial Block-Face Scanning Electron Microscopy to Enable High Resolution 3-D Nanohistology of Cells and Tissues. Microsc. Microanal. 16 (Suppl. 2), 1138–1139. 10.1017/S1431927610055170

[B24] Deerinck T. J. Shone T. M. Bushong E. A. Ramachandra R. Peltier S. T. Ellisman M. H. (2018). High-performance Serial Block-Face SEM of Nonconductive Biological Samples Enabled by Focal Gas Injection-Based Charge Compensation. J. Microsc. 270 (2), 142–149. 10.1111/jmi.12667 29194648 PMC5910240

[B25] Delevoye C. Heiligenstein X. Ripoll L. Gilles-Marsens F. Dennis M. K. Linares R. A. (2016). BLOC-1 Brings Together the Actin and Microtubule Cytoskeletons to Generate Recycling Endosomes. Curr. Biol. 26 (1), 1–13. 10.1016/j.cub.2015.11.020 26725201 PMC4713302

[B26] Dubin I. N. Sharp D. G. (1944). Comparison of the Morphology of Bacillus Megatherium with Light and Electron Microscopy. J. Bacteriol. 48 (3), 313–329. 10.1128/jb.48.3.313-329.1944 16560838 PMC373976

[B27] Dubochet J. Adrian M. Chang J.-J. Lepault J. McDowall A. W. (1987). Cryoelectron Microscopy of Vitrified Specimens. Cryotechniques Biol. Electron Microsc., 114–131. 10.1007/978-3-642-72815-0_5

[B28] Erickson P. A. Anderson D. H. Fisher S. K. (1987). Use of uranyl acetate *en bloc* to improve tissue preservation and labeling for post-embedding immunoelectron microscopy. J. Elec. Microsc. Tech. 5 (4), 303–314. 10.1002/jemt.1060050403

[B29] Faruqi A. R. McMullan G. (2011). Electronic Detectors for Electron Microscopy. Quart. Rev. Biophys. 44 (3), 357–390. 10.1017/S0033583511000035 21524337

[B30] Fermie J. Liv N. ten Brink C. van Donselaar E. G. Müller W. H. Schieber N. L. (2018). Single Organelle Dynamics Linked to 3D Structure by Correlative Live-Cell Imaging and 3D Electron Microscopy. Traffic 19 (5), 354–369. 10.1111/tra.12557 29451726

[B31] Fermie J. Zuidema W. Wolters A. H. G. Giepmans B. N. Hoogenboom J. P. (2020). “Rapid and Seamless Imaging of Biological Specimens Using a Novel Optical Scanning Transmission Electron Detector,” in European Microscopy Congress 2020.

[B32] Fu Z. Peng D. Zhang M. Xue F. Zhang R. He W. (2020). mEosEM Withstands Osmium Staining and Epon Embedding for Super-resolution CLEM. Nat. Methods 17 (1), 55–58. 10.1038/s41592-019-0613-6 31611693

[B33] Giddings T. H. (2003). Freeze-Substitution Protocols for Improved Visualization of Membranes in High-Pressure Frozen Samples. J. Microsc. 212 (1), 53–61. 10.1046/j.1365-2818.2003.01228.x 14516362

[B34] Giepmans B. N. G. Adams S. R. Ellisman M. H. Tsien R. Y. (2006). The Fluorescent Toolbox for Assessing Protein Location and Function. Science 312 (5771), 217–224. 10.1126/science.1124618 16614209

[B35] Giepmans B. N. G. Deerinck T. J. Smarr B. L. Jones Y. Z. Ellisman M. H. (2005). Correlated Light and Electron Microscopic Imaging of Multiple Endogenous Proteins Using Quantum Dots. Nat. Methods 2 (10), 743–749. 10.1038/nmeth791 16179920

[B36] Ginsburg H. Wolosin J. M. (1979). Effects of Uranyl Ions on Lipid Bilayer Membranes. Chem. Phys. Lipids 23 (2), 125–131. 10.1016/0009-3084(79)90040-9

[B37] Goetz J. G. Monduc F. Schwab Y. Vermot J. (2015). Using Correlative Light and Electron Microscopy to Study Zebrafish Vascular Morphogenesis. Tissue Morphog. Methods Protoc. 1189, 31–46. 10.1007/978-1-4939-1164-6_3 25245685

[B38] Hawes P. Netherton C. L. Mueller M. Wileman T. Monaghan P. (2007). Rapid Freeze-Substitution Preserves Membranes in High-Pressure Frozen Tissue Culture Cells. J. Microsc. 226 (2), 182–189. 10.1111/j.1365-2818.2007.01767.x 17444947

[B39] Hayat M. A. (1981). Fixation for Electron Microscopy. New York: Academic Press.

[B40] Hayat M. A. (1993). Stains and Cytochemical Methods. New York, USA and London, UK: Plenum Press.

[B41] Hayat M. A. (2002). Microscopy, Immunohistochemistry, and Antigen Retrieval Methods for Light and Electron Microscopy. New York, Boston, Dordrecht, London, Moscow: Kluwer Academic Publishers.

[B42] Heiligenstein X. de Beer M. Heiligenstein J. Eyraud F. Manet L. Schmitt F. (2021). HPM Live μ for a Full CLEM Workflow. Methods Cell Biol. 162, 115–149. 10.1016/bs.mcb.2020.10.022 33707009

[B43] Heiligenstein X. Heiligenstein J. Delevoye C. Hurbain I. Bardin S. Paul-Gilloteaux P. (2014). The CryoCapsule: Simplifying Correlative Light to Electron Microscopy. Traffic 15 (6), 700–716. 10.1111/tra.12164 24533564 PMC4064126

[B44] Hemelaar S. R. de Boer P. Chipaux M. Zuidema W. Hamoh T. Martinez F. P. (2017). Nanodiamonds as Multi-Purpose Labels for Microscopy. Sci. Rep. 7 (1), 720. 10.1038/s41598-017-00797-2 28389652 PMC5429637

[B45] Hippe-Sanwald S. (1993). Impact of Freeze Substitution on Biological Electron Microscopy. Microsc. Res. Tech. 24 (5), 400–422. 10.1002/jemt.1070240506 8318724

[B46] Hodgson L. Nam D. Mantell J. Achim A. Verkade P. (2014). Retracing in Correlative Light Electron Microscopy: Where is my Object of Interest? Methods Cell Biol. 124, 1–21. 10.1016/b978-0-12-801075-4.00001-x 25287834

[B47] Höhn K. Fuchs J. Fröber A. Kirmse R. Glass B. Anders-össwein M. (2015). Preservation of Protein Fluorescence in Embedded Human Dendritic Cells for Targeted 3D Light and Electron Microscopy. J. Microsc. 259 (2), 121–128. 10.1111/jmi.12230 25786567 PMC4757415

[B48] Hosogi N. Nishioka H. Nakakoshi M. (2015). Evaluation of Lanthanide Salts as Alternative Stains to Uranyl Acetate. Microsc. (Tokyo) 64 (6), 429–435. 10.1093/jmicro/dfv054 26374081

[B49] Howes S. C. Koning R. I. Koster A. J. (2018). Correlative Microscopy for Structural Microbiology. Curr. Opin. Microbiol. 43, 132–138. 10.1016/j.mib.2018.01.009 29414444

[B50] Humbel B. M. Schwarz H. (1989). “Freeze-substitution for Immunochemistry,” in Immuno-Gold Labeling in Cell Biology. Editors Verkleij A. J. Leunissen J. L. M. (Boca Raton, FL: CRC Press), 115–134.

[B51] Inaga S. Katsumoto T. Tanaka K. Kameie T. Nakane H. Naguro T. (2007). Platinum Blue as an Alternative to Uranyl Acetate for Staining in Transmission Electron Microscopy. Archives Histology Cytol. 70 (1), 43–49. 10.1679/aohc.70.43 17558143

[B52] Jahn K. A. Barton D. A. Kobayashi K. Ratinac K. R. Overall R. L. Braet F. (2012). Correlative Microscopy: Providing New Understanding in the Biomedical and Plant Sciences. Micron 43 (5), 565–582. 10.1016/j.micron.2011.12.004 22244153

[B53] Jansen J. Reimer K. C. Nagai J. S. Varghese F. S. Overheul G. J. de Beer M. (2021). SARS-CoV-2 Infects the Human Kidney and Drives Fibrosis in Kidney Organoids. Cell Stem Cell 29, 217–231. 10.1016/j.stem.2021.12.010 35032430 PMC8709832

[B54] Johnson E. Seiradake E. Jones E. Y. Davis I. Grünewald K. Kaufmann R. (2015). Correlative In-Resin Super-resolution and Electron Microscopy Using Standard Fluorescent Proteins. Sci. Rep. 5, 9583. 10.1038/srep09583 25823571 PMC4379466

[B55] Karnovsky M. J. (1965). A Formaldehyde-Glutaraldehyde Fixative of High Osmolality for Use in Electron Microscopy. J. Cell Biol. 27 (2), A137–A138.

[B56] Karreman M. A. Hyenne V. Schwab Y. Goetz J. G. (2016). Intravital Correlative Microscopy: Imaging Life at the Nanoscale. Trends Cell Biol. 26 (11), 848–863. 10.1016/j.tcb.2016.07.003 27515435

[B57] Karreman M. A. Mercier L. Schieber N. L. Shibue T. Schwab Y. Goetz J. G. (2014). Correlating Intravital Multi-Photon Microscopy to 3D Electron Microscopy of Invading Tumor Cells Using Anatomical Reference Points. Plos One 9 (12), e114448. 10.1371/journal.pone.0114448 25479106 PMC4257674

[B58] Karreman M. A. Ruthensteiner B. Mercier L. Schieber N. L. Solecki G. Winkler F. (2017). Find Your Way with X-Ray: Using MicroCT to Correlate *in Vivo* Imaging With 3D Electron Microscopy. Methods Cell Biol. 140, 277–301. 10.1016/bs.mcb.2017.03.006 28528637

[B59] Keene D. R. Tufa S. F. Lunstrum G. P. Holden P. Horton W. A. (2008). Confocal/TEM Overlay Microscopy: a Simple Method for Correlating Confocal and Electron Microscopy of Cells Expressing GFP/YFP Fusion Proteins. Microsc. Microanal. 14 (4), 342–348. 10.1017/s1431927608080306 18598569

[B60] Keevend K. Krummenacher R. Kungas E. Gerken L. R. H. Gogos A. Stiefel M. (2020). Correlative Cathodoluminescence Electron Microscopy: Immunolabeling Using Rare-Earth Element Doped Nanoparticles. Small 16 (44), 2004615. 10.1002/smll.202004615 33090693

[B61] Kellenberger E. Dürrenberger M. Villiger W. Carlemalm E. Wurtz M. (1987). The Efficiency of Immunolabel on Lowicryl Sections Compared to Theoretical Predictions. J. Histochem Cytochem. 35 (9), 959–969. 10.1177/35.9.3302020 3302020

[B62] Kellenberger E. (1987). “The Response of Biological Macromolecules and Supramolecular Structures to the Physics of Specimen Cryopreparation,” in Cryotechniques in Biological Electron Microscopy. Editors Steinbrecht R. A. Zierold K. (Berlin, Heidelberg: Springer Berlin Heidelberg), 35–63. 10.1007/978-3-642-72815-0_2

[B63] Knott G. W. Holtmaat A. Trachtenberg J. T. Svoboda K. Welker E. (2009). A Protocol for Preparing GFP-Labeled Neurons Previously Imaged *In Vivo* and in Slice Preparations for Light and Electron Microscopic Analysis. Nat. Protoc. 4 (8), 1145–1156. 10.1038/nprot.2009.114 19617886

[B64] Kolotuev I. Bumbarger D. J. Labouesse M. Schwab Y. (2012). Targeted Ultramicrotomy: A Valuable Tool for Correlated Light and Electron Microscopy of Small Model Organisms. Methods Cell Biol. 111, 203–222. 10.1016/b978-0-12-416026-2.00011-x 22857930

[B65] Kopek B. G. Paez-Segala M. G. Shtengel G. Sochacki K. A. Sun M. G. Wang Y. (2017). Diverse Protocols for Correlative Super-resolution Fluorescence Imaging and Electron Microscopy of Chemically Fixed Samples. Nat. Protoc. 12 (5), 916–946. 10.1038/nprot.2017.017 28384138 PMC5514615

[B66] Kremer A. Lippens S. Bartunkova S. Asselbergh B. Blanpain C. Fendrych M. (2015). Developing 3D SEM in a Broad Biological Context. J. Microsc. 259, 80–96. 10.1111/jmi.12211 25623622 PMC4670703

[B67] Kuipers J. Giepmans B. N. G. (2020). Neodymium as an Alternative Contrast for Uranium in Electron Microscopy. Histochem Cell Biol. 153 (4), 271–277. 10.1007/s00418-020-01846-0 32008069 PMC7160090

[B68] Kukulski W. Schorb M. Kaksonen M. Briggs J. A. G. (2012). Plasma Membrane Reshaping during Endocytosis Is Revealed by Time-Resolved Electron Tomography. Cell 150 (3), 508–520. 10.1016/j.cell.2012.05.046 22863005

[B69] Kukulski W. Schorb M. Welsch S. Picco A. Kaksonen M. Briggs J. A. G. (2011). Correlated Fluorescence and 3D Electron Microscopy with High Sensitivity and Spatial Precision. J. Cell Biol. 192 (1), 111–119. 10.1083/jcb.201009037 21200030 PMC3019550

[B70] Lane R. Vos Y. Wolters A. H. G. Kessel L. v. Chen S. E. Liv N. (2021). Optimization of Negative Stage Bias Potential for Faster Imaging in Large-Scale Electron Microscopy. J. Struct. Biol. X 5, 100046. 10.1016/j.yjsbx.2021.100046 33763642 PMC7973379

[B71] Lane R. Wolters A. H. G. Giepmans B. N. G. Hoogenboom J. P. (2022). Integrated Array Tomography for 3D Correlative Light and Electron Microscopy. Front. Mol. Biosci. 8, 822232. 10.3389/fmolb.2021.822232 35127826 PMC8809480

[B72] Lemercier N. Middel V. Hentsch D. Taubert S. Takamiya M. Beil T. (2017). Microtome-Integrated Microscope System for High Sensitivity Tracking of In-Resin Fluorescence in Blocks and Ultrathin Sections for Correlative Microscopy. Sci. Rep. 7, 11. 10.1038/s41598-017-13348-6 29051533 PMC5648784

[B73] Lepock J. R. (1997). Protein Denaturation During Heat Shock. Adv. Mol. Cell Biol. 19, 223–259. 10.1016/s1569-2558(08)60079-x

[B74] Levin B. D. A. (2021). Direct Detectors and Their Applications in Electron Microscopy for Materials Science. J. Phys. Mat. 4 (4), 042005. 10.1088/2515-7639/ac0ff9

[B75] Levitt M. F. Gaudino M. (1950). Measurement of Body Water Compartments. Am. J. Med. 9 (2), 208–215. 10.1016/0002-9343(50)90024-6 15432468

[B76] Ling R. (2004). Introduction. Physiol. Chem. Phys. Med. NMR 36 (1), 1–19. 10.1016/b978-155860936-5/50001-5 15789970

[B77] Liv N. Zonnevylle A. C. Narvaez A. C. Effting A. P. J. Voorneveld P. W. Lucas M. S. (2013). Simultaneous Correlative Scanning Electron and High-NA Fluorescence Microscopy. Plos One 8 (2), e55707. 10.1371/journal.pone.0055707 23409024 PMC3568124

[B78] Loussert Fonta C. Humbel B. M. (2015). Correlative Microscopy. Archives Biochem. Biophysics 581, 98–110. 10.1016/j.abb.2015.05.017 26072116

[B79] Luby-Phelps K. Ning G. Fogerty J. Besharse J. C. (2003). Visualization of Identified GFP-Expressing Cells by Light and Electron Microscopy. J. Histochem Cytochem. 51 (3), 271–274. 10.1177/002215540305100301 12588954

[B80] Lucas F. Günthert M. Wepf R. Lucas M. S. (2017). Towards Improved In-Resin Fluorescence Protocols for CLEM: A Method to Monitor Fluorescence Quenching during Sample Preparation.

[B81] Lucas M. S. Günthert M. Bittermann A. G. de Marco A. Wepf R. (2017). Correlation of Live-Cell Imaging with Volume Scanning Electron Microscopy. Methods Cell Biol. 140, 123–148. 10.1016/bs.mcb.2017.03.001 28528630

[B82] Lucas M. S. Günthert M. Gasser P. Lucas F. Wepf R. (2012). Bridging Microscopes: 3D Correlative Light and Scanning Electron Microscopy of Complex Biological Structures. Methods Cell Biol. 111, 325–356. 10.1016/b978-0-12-416026-2.00017-0 22857936

[B83] Luft J. H. (1961). Improvements in Epoxy Resin Embedding Methods. J. Biophysical Biochem. Cytol. 9 (2), 409–414. 10.1083/jcb.9.2.409 PMC2224998 13764136

[B84] Matsko N. Mueller M. (2005). Epoxy Resin as Fixative during Freeze-Substitution. J. Struct. Biol. 152 (2), 92–103. 10.1016/j.jsb.2005.07.005 16214372

[B85] McDonald K. (2007). Cryopreparation Methods for Electron Microscopy of Selected Model Systems. Methods Cell Biol. 79, 23–56. 10.1016/s0091-679x(06)79002-1 17327151

[B86] McDonald K. L. Webb R. I. (2011). Freeze Substitution in 3 hours or Less. J. Microsc. 243 (3), 227–233. 10.1111/j.1365-2818.2011.03526.x 21827481

[B87] Micheva K. D. O'Rourke N. Busse B. Smith S. J. (2010). Array Tomography: Immunostaining and Antibody Elution. Cold Spring Harb. Protoc. 2010 (11), pdb. pdb prot5525. 10.1101/pdb.prot5525 21041398

[B88] Micheva K. D. Smith S. J. (2007). Array Tomography: A New Tool for Imaging the Molecular Architecture and Ultrastructure of Neural Circuits. Neuron 55 (1), 25–36. 10.1016/j.neuron.2007.06.014 17610815 PMC2080672

[B89] Mironov A. A. Polishchuk R. S. Luini A. (2000). Visualizing Membrane Traffic *In Vivo* by Combined Video Fluorescence and 3D Electron Microscopy. Trends Cell Biol. 10 (8), 349–353. 10.1016/s0962-8924(00)01787-6 10884688

[B90] Monaghan P. Perusinghe N. Muller M. (1998). High-pressure Freezing for Immunocytochemistry. J. Microsc. 192 (3), 248–258. 10.1046/j.1365-2818.1998.00387.x 9923417

[B91] Moor H. (1973). “Cryotechnology for the Structural Analysis of Biological Material,” in Freeze-etching Techniques and Applications. Editors Benedetti E. L. Favard P. (Paris: Societe Franqaise de Microscopic Electronique).

[B92] Murk J. L. A. N. Posthuma G. Koster A. J. Geuze H. J. Verkleij A. J. Kleijmeer M. J. (2003). Influence of Aldehyde Fixation on the Morphology of Endosomes and Lysosomes: Quantitative Analysis and Electron Tomography. J. Microsc. 212 (1), 81–90. 10.1046/j.1365-2818.2003.01238.x 14516365

[B93] Nakakoshi M. Nishioka H. Katayama E. (2011). New Versatile Staining Reagents for Biological Transmission Electron Microscopy that Substitute for Uranyl Acetate. J. Electron Microsc. 60 (6), 401–407. 10.1093/jmicro/dfr084 22146677

[B94] Newman G. R. Hobot J. A. (1993). Resin Microscopy and On-Section Immunocytochemistry. Berlin, Heidelberg: Springer.

[B95] Nixon S. J. Webb R. I. Floetenmeyer M. Schieber N. Lo H. P. Parton R. G. (2009). A Single Method for Cryofixation and Correlative Light, Electron Microscopy and Tomography of Zebrafish Embryos. Traffic 10 (2), 131–136. 10.1111/j.1600-0854.2008.00859.x 19054388

[B96] Oberti D. Kirschmann M. A. Hahnloser R. H. (2010). Correlative Microscopy of Densely Labeled Projection Neurons Using Neural Tracers. Front. Neuroanat. 4, 24. 10.3389/fnana.2010.00024 20676237 PMC2912169

[B97] Paez-Segala M. G. Sun M. G. Shtengel G. Viswanathan S. Baird M. A. Macklin J. J. (2015). Fixation-resistant Photoactivatable Fluorescent Proteins for CLEM. Nat. Methods 12 (3), 215–218. 10.1038/nmeth.3225 25581799 PMC4344411

[B98] Peddie C. J. Blight K. Wilson E. Melia C. Marrison J. Carzaniga R. (2014). Correlative and Integrated Light and Electron Microscopy of In-Resin GFP Fluorescence, Used to Localise Diacylglycerol in Mammalian Cells. Ultramicroscopy 143, 3–14. 10.1016/j.ultramic.2014.02.001 24637200 PMC4045205

[B99] Peddie C. J. Collinson L. M. (2014). Exploring the Third Dimension: Volume Electron Microscopy Comes of Age. Micron 61, 9–19. 10.1016/j.micron.2014.01.009 24792442

[B100] Peddie C. J. Domart M.-C. Snetkov X. O'Toole P. Larijani B. Way M. (2017). Correlative Super-resolution Fluorescence and Electron Microscopy Using Conventional Fluorescent Proteins In Vacuo. J. Struct. Biol. 199 (2), 120–131. 10.1016/j.jsb.2017.05.013 28576556 PMC5531056

[B101] Polishchuk R. S. Polishchuk E. V. Marra P. Alberti S. Buccione R. Luini A. (2000). Correlative Light-Electron Microscopy Reveals the Tubular-Saccular Ultrastructure of Carriers Operating between Golgi Apparatus and Plasma Membrane. J. Cell Biol. 148 (1), 45–58. 10.1083/jcb.148.1.45 10629217 PMC2156208

[B102] Prangsma J. C. Molenaar R. van Weeren L. Bindels D. S. Haarbosch L. Stouthamer J. (2020). Quantitative Determination of Dark Chromophore Population Explains the Apparent Low Quantum Yield of Red Fluorescent Proteins. J. Phys. Chem. B 124 (8), 1383–1391. 10.1021/acs.jpcb.9b10396 32011884 PMC7049984

[B103] Prior D. A. M. Oparka K. J. Roberts I. M. (1999). En Blocoptical Sectioning of Resin-Embedded Specimens Using a Confocal Laser Scanning Microscope. J. Microsc. 193 (1), 20–27. 10.1046/j.1365-2818.1999.00433.x 12558684

[B104] Reinoso R. F. Telfer B. A. Rowland M. (1997). Tissue Water Content in Rats Measured by Desiccation. J. Pharmacol. Toxicol. Methods 38 (2), 87–92. 10.1016/S1056-8719(97)00053-1 9403779

[B105] Riehle U. Hoechli M. (1973). “The Theory and Technique of High Pressure Freezing,” in Freeze-etching Techniques and Applications. Editors Benedetti E. L. Favard P. (Paris: Societe Francaise de Microscopic Electronique), 11–19.

[B106] Ripoll L. Heiligenstein X. Hurbain I. Domingues L. Figon F. Petersen K. J. (2018). Myosin VI and Branched Actin Filaments Mediate Membrane Constriction and Fission of Melanosomal Tubule Carriers. J. Cell Biol. 217 (8), 2709–2726. 10.1083/jcb.201709055 29875258 PMC6080934

[B107] Rizzo N. W. Duncan K. E. Bourett T. M. Howard R. J. (2016). Backscattered Electron SEM Imaging of Resin Sections from Plant Specimens: Observation of Histological to Subcellular Structure and CLEM. J. Microsc. 263 (2), 142–147. 10.1111/jmi.12373 26708578

[B108] Robertson D. Monaghan P. Clarke C. Atherton A. J. (1992). An Appraisal of Low-Temperature Embedding by Progressive Lowering of Temperature into Lowicryl HM20 for Immunocytochemical Studies. J. Microsc. 168, 85–100. 10.1111/j.1365-2818.1992.tb03253.x 1447755

[B109] Sabatini D. D. Bensch K. Barrnett R. J. (1963). Cytochemistry and Electron Microscopy. The Preservation of Cellular Ultrastructure and Enzymatic Activity by Aldehyde Fixation. J. Cell Biol. 17 (1), 19–58. 10.1083/jcb.17.1.19 13975866 PMC2106262

[B110] Sato S. Sasaki Y. Adachi A. Dai W. Liu X.-L. Namimatsu S. (2003). Use of Oolong Tea Extract (OTE) for Elastin Staining and Enhancement in Ultrathin Sections. Med. Electron Microsc. 36 (3), 179–182. 10.1007/s00795-003-0216-1 14505062

[B111] Scher N. Avinoam O. (2021). 50 Shades of CLEM: How to Choose the Right Approach for You. Methods Cell Biol. 162, 1–11. 10.1016/bs.mcb.2020.08.001 33707008

[B112] Sims P. Albrecht R. Pawley J. B. Centonze V. Deerinck T. Hardin J. (2006). When Light Microscope Resolution Is Not Enough:Correlational Light Microscopy and Electron Microscopy. Handb. Biol. Confocal Microsc., 846–860. 10.1007/978-0-387-45524-2_49

[B113] Su Y. Nykanen M. Jahn K. A. Whan R. Cantrill L. Soon L. L. (2010). Multi-dimensional Correlative Imaging of Subcellular Events: Combining the Strengths of Light and Electron Microscopy. Biophys. Rev. 2 (3), 121–135. 10.1007/s12551-010-0035-2 28510069 PMC5418370

[B114] Tagliaferro P. Tandler C. J. Ramos A. J. Pecci Saavedra J. Brusco A. (1997). Immunofluorescence and Glutaraldehyde Fixation. A New Procedure Based on the Schiff-Quenching Method. J. Neurosci. Methods 77 (2), 191–197. 10.1016/s0165-0270(97)00126-x 9489897

[B115] Timmermans F. J. Otto C. (2015). Contributed Review: Review of Integrated Correlative Light and Electron Microscopy. Rev. Sci. Instrum. 86 (1), 011501. 10.1063/1.4905434 25638065

[B116] Titze B. Genoud C. (2016). Volume Scanning Electron Microscopy for Imaging Biological Ultrastructure. Biol. Cell 108 (11), 307–323. 10.1111/boc.201600024 27432264

[B117] Tranfield E. M. Heiligenstein X. Peristere I. Antony C. (2014). Correlative Light and Electron Microscopy for a Free-Floating Spindle in *Xenopus laevis* Egg Extracts. Methods Cell Biol. 124, 111–128. 10.1016/b978-0-12-801075-4.00006-9 25287839

[B118] Tuijtel M. W. Mulder A. A. Posthuma C. C. van der Hoeven B. Koster A. J. Bárcena M. (2017). Inducing Fluorescence of Uranyl Acetate as a Dual-Purpose Contrast Agent for Correlative Light-Electron Microscopy with Nanometre Precision. Sci. Rep. 7 (1), 10442. 10.1038/s41598-017-10905-x 28874723 PMC5585351

[B119] van Rijnsoever C. Oorschot V. Klumperman J. (2008). Correlative Light-Electron Microscopy (CLEM) Combining Live-Cell Imaging and Immunolabeling of Ultrathin Cryosections. Nat. Methods 5 (11), 973–980. 10.1038/nmeth.1263 18974735

[B120] Verkade P. (2008). Moving EM: the Rapid Transfer System as a New Tool for Correlative Light and Electron Microscopy and High Throughput for High-Pressure Freezing. J. Microsc. 230 (2), 317–328. 10.1111/j.1365-2818.2008.01989.x 18445162

[B121] Vos Y. Lane R. I. Peddie C. J. Wolters A. H. G. Hoogenboom J. P. (2020). Retarding Field Integrated Fluorescence and Electron Microscope. Microsc. Microanal. 27, 109–120. 10.1017/S1431927620024745 33349285

[B122] Wacker I. Chockley P. Bartels C. Spomer W. Hofmann A. Gengenbach U. (2015). Array Tomography: Characterizing FAC-Sorted Populations of Zebrafish Immune Cells by Their 3D Ultrastructure. J. Microsc. 259 (2), 105–113. 10.1111/jmi.12223 25611576 PMC4670706

[B123] Wacker I. Schroeder R. R. (2013). Array Tomography. J. Microsc. 252 (2), 93–99. 10.1111/jmi.12087 24111814

[B124] Wall K. P. Dillon R. Knowles M. K. (2015). Fluorescence Quantum Yield Measurements of Fluorescent Proteins: A Laboratory Experiment for a Biochemistry or Molecular Biophysics Laboratory Course. Biochem. Mol. Biol. Educ. 43 (1), 52–59. 10.1002/bmb.20837 25395254

[B125] Walther P. Ziegler A. (2002). Freeze Substitution of High-Pressure Frozen Samples: the Visibility of Biological Membranes Is Improved when the Substitution Medium Contains Water. J. Microsc. 208 (1), 3–10. 10.1046/j.1365-2818.2002.01064.x 12366592

[B126] Ward W. W. (2006). Biochemical and Physical Properties of Green Fluorescent Protein. Green Fluoresc. Protein Prop. Appl. Protoc. 47, 39–65. 16335709

[B127] Watanabe S. Punge A. Hollopeter G. Willig K. I. Hobson R. J. Davis M. W. (2011). Protein Localization in Electron Micrographs Using Fluorescence Nanoscopy. Nat. Methods 8 (1), 80–84. 10.1038/nmeth.1537 21102453 PMC3059187

[B128] Weibull C. Christiansson A. (1986). Extraction of Proteins and Membrane Lipids during Low Temperature Embedding of Biological Material for Electron Microscopy. J. Microscopy-Oxford 142, 79–86. 10.1111/j.1365-2818.1986.tb02739.x 3712423

[B129] Wild P. Schraner E. M. Adler H. Humbel B. M. (2001). Enhanced Resolution of Membranes in Cultured Cells by Cryoimmobilization and Freeze-Substitution. Microsc. Res. Tech. 53 (4), 313–321. 10.1002/jemt.1098 11340677

[B130] Wilke K. Wick K. Keil F. J. Wittern K. P. Wepf R. Biel S. S. (2008). A Strategy for Correlative Microscopy of Large Skin Samples: towards a Holistic View of Axillary Skin Complexity. Exp. Dermatol 17 (1), 73–81. 10.1111/j.1600-0625.2007.00635.x 18005049

[B131] Yahav T. Maimon T. Grossman E. Dahan I. Medalia O. (2011). Cryo-electron Tomography: Gaining Insight into Cellular Processes by Structural Approaches. Curr. Opin. Struct. Biol. 21, 670–677. 10.1016/j.sbi.2011.07.004 21813274

[B132] Yamaguchi K. Suzuki K. i. Tanaka K. (2010). Examination of Electron Stains as a Substitute for Uranyl Acetate for the Ultrathin Sections of Bacterial Cells. J. Electron Microsc. 59 (2), 113–118. 10.1093/jmicro/dfp045 19767626

[B133] Zhou H. Gang Y. Chen S. Wang Y. Xiong Y. Li L. (2017). Development of a Neutral Embedding Resin for Optical Imaging of Fluorescently Labeled Biological Tissue. J. Biomed. Opt. 22 (10), 1–7. 10.1117/1.JBO.22.10.106015 29076308

